# Global trends in antibiotic consumption during 2016–2023 and future projections through 2030

**DOI:** 10.1073/pnas.2411919121

**Published:** 2024-11-18

**Authors:** Eili Y. Klein, Isabella Impalli, Suprena Poleon, Philippe Denoel, Mariateresa Cipriano, Thomas P. Van Boeckel, Simone Pecetta, David E. Bloom, Arindam Nandi

**Affiliations:** ^a^One Health Trust, Washington, DC 20015; ^b^Department of Emergency Medicine, Johns Hopkins School of Medicine, Baltimore, MD 21287; ^c^GSK, Vaccines R&D, Rixensart 1330, Belgium; ^d^GSK, Vaccines R&D, Siena 53100, Italy; ^e^One Health Institute, University of Zürich, Zürich 8057, Switzerland; ^f^Spatial Epidemiology Lab, Université Libre de Bruxelles, Brussels B-1050, Belgium; ^g^Harvard T. H. Chan School of Public Health, Boston, MA 02115; ^h^The Population Council, New York, NY 10017

**Keywords:** antibiotic resistance, AMR, global public health, antimicrobial resistance

## Abstract

Antibiotic resistance is a global public health threat. Many factors contribute to this issue, with human antibiotic consumption being significant among them. Analyzing trends and patterns in consumption can aid in developing policies to mitigate the burden of antimicrobial resistance and global disparities in access to antibiotics. Using pharmaceutical sales data licensed from IQVIA, we estimate national-level trends in antibiotic consumption in 67 countries during 2016–2023 and analyze the effects of economic growth and the COVID-19 pandemic. Finally, we estimate global human consumption and project growth through 2030 assuming current trends. We find that estimated antibiotic consumption in reported countries increased 16.3% from 29.5 to 34.3 billion defined daily doses (DDDs) from 2016 to 2023, reflecting a 10.6% increase in the consumption rate from 13.7 to 15.2 DDDs per 1,000 inhabitants per day. Increases were most pronounced in upper-middle- and lower-middle-income countries. While the COVID-19 pandemic significantly reduced consumption globally, this was most pronounced in high-income countries, and in these countries, reductions in antibiotic use in 2020 were sharper, and lasted longer, than in other countries. By 2030, we project that, without reductions in rapidly developing nations, such as investments to improve infrastructure, particularly water and sanitation, along with improved access to vaccination, global antibiotic consumption will increase by 52.3% from an estimated 49.3 billion in 2023 to 75.1 billion DDDs.

## Significance

Antimicrobial resistance is a pressing global health challenge driven by human antibiotic consumption, among other factors. In this report, we investigate trends in human antibiotic consumption in 67 countries from 2016 to 2023, focusing on changes in consumption during the COVID-19 pandemic. We found that global antibiotic consumption declined during the COVID-19 pandemic, but rebounded thereafter, particularly in middle-income countries. While our estimate of 49.3 billion defined daily doses for total global use is lower than previous forecasts, reductions associated with the pandemic make it challenging to determine whether attempts to curb antibiotic use over the past decade have been effective. Moreover, postpandemic increases are worrying in their implications for the future trajectory of use.

Antibiotic resistance is a critical global health challenge. Estimates of the burden of resistance suggest that nearly 5 million deaths were associated with bacterial resistance to antibiotics in 2019 (1). Notably, low-income countries, particularly in sub-Saharan Africa, had the highest rates of mortality associated with bacterial resistance despite lower consumption rates. However, routine testing for resistance is relatively rare in Africa, which may underrepresent the true correlation between resistance and mortality. While resistance is driven by overuse and misuse of antibiotics in humans, animals, and agriculture, as well as poor infection prevention and control, human antibiotic use is a major driver (2, 3). Global human antibiotic consumption increased by 65% between 2000 and 2015, driven primarily by lower-middle-income countries (LMICs) and the rise in gross domestic product (GDP) in LMICs, though rates remained significantly higher in many high-income countries (HICs) (4, 5).

Since penicillin became widely available in the 1940s (6), antibiotics have played an indispensable role in reducing morbidity and mortality from both common ailments, such as streptococcal infections, and life-threatening conditions, like sepsis. However, while antibiotics have played a crucial role in reducing morbidity and mortality from bacterial infections (7, 8), the largest gains in life expectancy in HICs largely occurred prior to the introduction of antibiotics with the implementation of public health measures including improved sanitation and sewage management, public water treatment and food inspection, and surveillance and control of infectious diseases, including vaccination (9). In many LMICs, antibiotics are used to decrease the morbidity and mortality of illnesses that are directly attributable to lack of universal access to clean water and improved sanitation and hygiene (10). However, relying heavily on antibiotics in lieu of improving sanitation and other public health measures can exacerbate the problem of antibiotic resistance.

Surveillance of antibiotic consumption provides a foundation for improving antibiotic stewardship. Identifying trends in use can help to tailor educational and vaccination campaigns, policy recommendations, and clinical guidelines to the unique challenges of each region or country. This is especially important in LMICs, which are often forced to grapple with the juxtaposition of limited access to essential antibiotics and the indiscriminate or inappropriate use of these agents (4, 11). In HICs, which until now have had some of the highest per capita antibiotic consumption rates, inappropriate use of antibiotics, such as for influenza-like illnesses and other viral infections, continues to be a major challenge (4, 5). Monitoring consumption patterns can provide insights into these disparities, guiding equitable distribution and accessibility initiatives. Furthermore, as antibiotic resistance knows no borders, ensuring robust surveillance in LMICs, which might lack the resources or infrastructure for such efforts, is not only crucial for their local populations but also forms a linchpin in the global strategy to combat the escalating threat of antibiotic resistance.

Here, we used antibiotic sales data from IQVIA MIDAS and conducted an analysis of trends in antibiotic consumption, focusing on the differences in consumption associated with World Bank income classification throughout the study period 2016–2023. Additionally, we quantify the impact of the COVID-19 pandemic on antibiotic use, where despite several studies finding low rates of bacterial coinfection, antibiotics were continually prescribed to COVID-19 patients at high rates (12–16). Our study has important implications for the growing burden of multi-drug resistant (MDR) bacteria in many countries. The direct link between increased antibiotic consumption and prevalence of MDR bacteria (3, 17) is of significant concern globally.

## Results

Total antibiotic consumption for countries with available data (n = 67) increased by 16.3% between 2016 and 2023, from 29.5 to 34.3 billion defined daily doses (DDDs), and the antibiotic consumption rate across these countries increased 10.6% from 13.7 to 15.2 DDDs per 1,000 inhabitants per day. The mean antibiotic consumption rate across those 67 countries increased by 5.5% from 19.5 DDDs per 1,000 inhabitants per day to 20.5, and the median antibiotic consumption rate increased by 1.7% from 18.5 to 18.8 DDDs per 1,000 inhabitants per day.

**Fig. 1.**
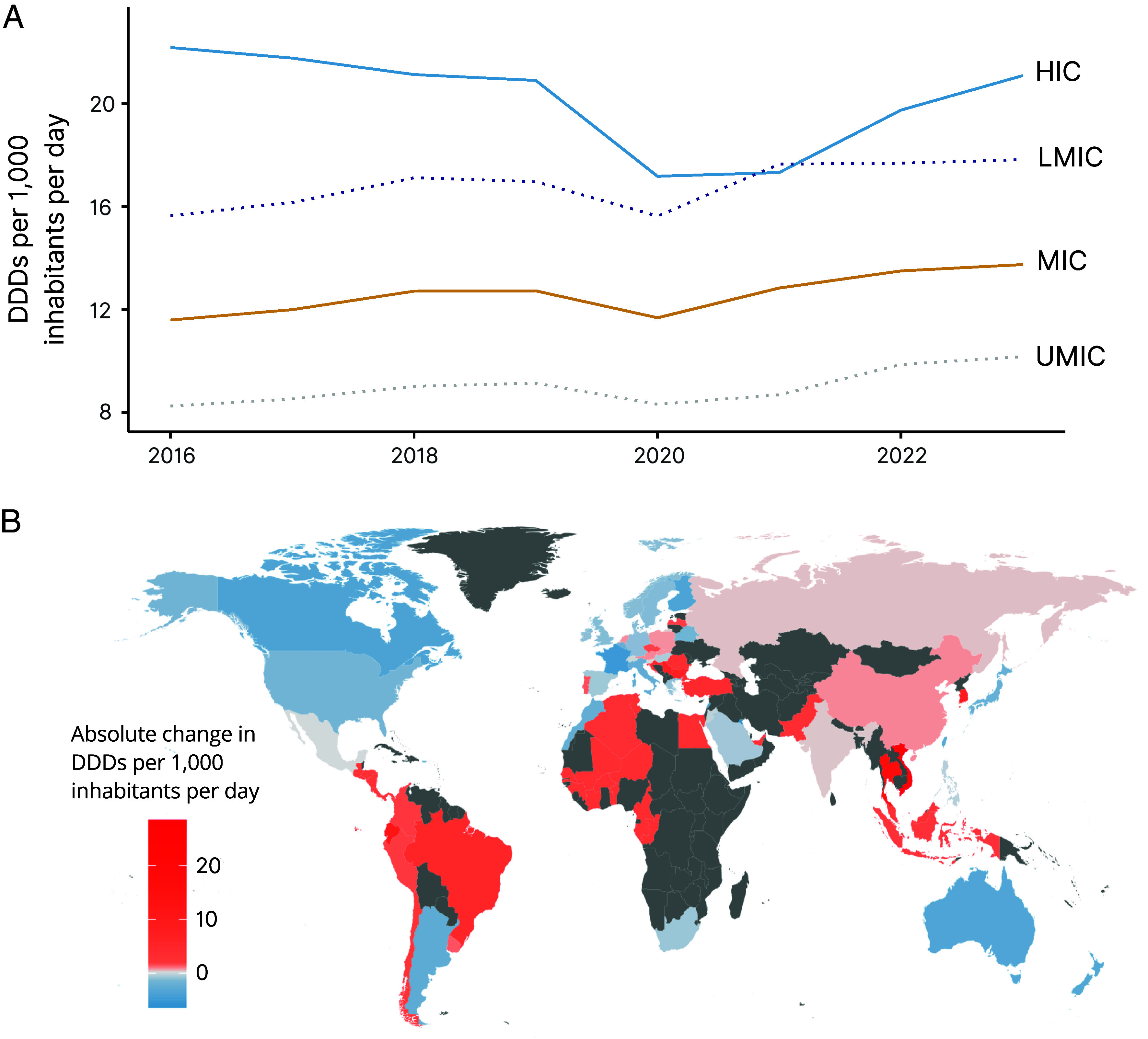
Change in global antibiotic consumption by country and country income classification, 2016–2023. (*A*) Yearly antibiotic consumption rate, measured in DDDs per 1,000 inhabitants per day, by country income classification. (*B*) Absolute change in antibiotic consumption rate between 2016 and 2023 by country in DDDs per 1,000 inhabitants per day. Countries in gray have no data in the database. Country income classifications noted as LMIC = lower-middle-income countries, MIC = middle-income countries, UMIC = upper-middle-income countries, HIC = high-income countries. Data Source: Based on IQVIA MIDAS^®^ sales data for period 2016–2023. Copyright IQVIA. All rights reserved.

Increases in consumption varied across income groups. For this analysis, the income groups used as defined by the World Bank include middle-income countries (MICs), which are broken out into LMICs (n = 11) and upper-middle-income countries (UMICs, n = 17) where appropriate, and HICs (n = 39). Between 2016 and 2019, consumption rates increased in MICs (9.8%) while decreasing in HICs (−5.8%) (Fig. 1). The COVID-19 pandemic significantly reduced consumption across all income groups; this was most pronounced in HICs (−17.8%) in 2020. However, MICs saw rapid increases in 2021, and in that year, LMICs led HICs in consumption rate, at 17.7 and 17.3 DDDs per 1,000 inhabitants per day, respectively. For the entire study period 2016–2023, the consumption rate in MICs increased by 18.6% (23.4% in UMICs and 14.0% in LMICs), and the consumption rate in HICs decreased by 4.9%. Interrupted time series analysis (ITSA) results indicated that while consumption in HICs was decreasing at a rate of 0.34 (SE = 0.05) DDDs per 1,000 inhabitants per day before the onset of the pandemic, it fell by 5.73 DDDs per 1,000 inhabitants per day in 2020 and then increased at a rate of 1.39 DDDs per 1,000 inhabitants per day in the postpandemic period (*SI Appendix*, Fig. S1 and Table S1). In MICs, prepandemic trends were increasing (0.32 DDDs per 1,000 inhabitants per day, SE = 0.00) but decreased 1.50 DDDs per 1,000 inhabitants per day in 2020 and then increased at a faster rate after the pandemic (0.70 DDDs per 1,000 inhabitants per day annually) than before.

**Fig. 2.**
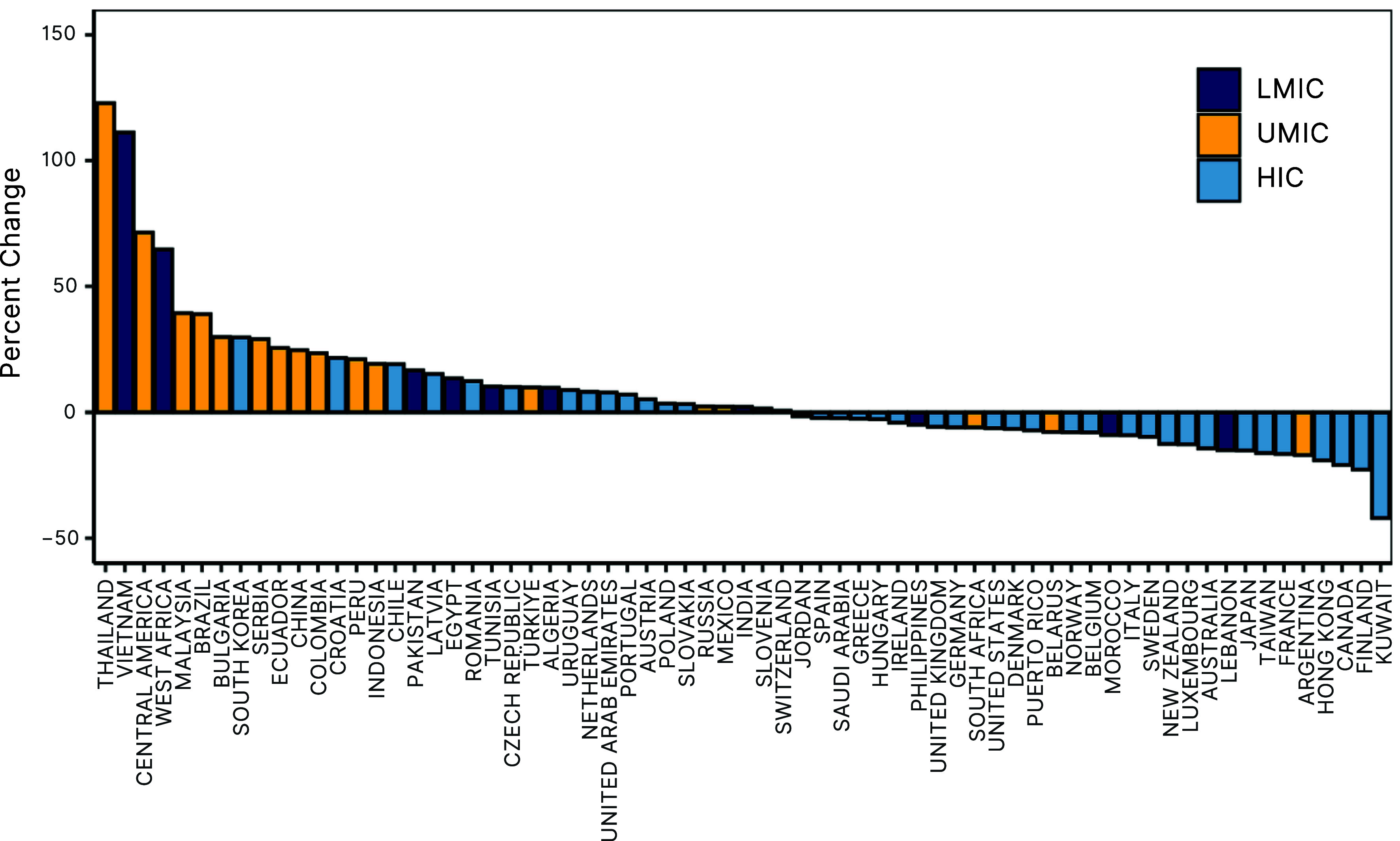
Change in each antibiotic consumption rate, by country. Results are the percentage change between each country’s consumption rate in 2016–2023. Country income classifications noted as LMIC = lower-middle-income countries, UMIC = upper-middle-income countries, HIC = high-income countries. Data Source: Based on IQVIA MIDAS^®^ sales data for period 2016–2023. Copyright IQVIA. All rights reserved.

The top five largest percentage increases over the study period occurred in MICs (Fig. 2). The most significant consumption increases in LMICs were observed in Vietnam, where the consumption rate more than doubled from 25.6 to 54.0 DDDs per 1,000 inhabitants per day (111.2%), followed by West Africa (5.7 to 9.4, 64.8%). The most significant increases in UMICs were observed in Thailand, which increased from 12.7 to 28.3 DDDs per 1,000 inhabitants per day (122.8%) followed by Central America (4.0 to 6.8, 71.6%) and Malaysia (7.6 to 10.6, 39.6%). Three of the top ten countries with the highest consumption rates in 2023 were LMIC countries (Vietnam, Algeria, and Tunisia), compared with two in 2016 (Tunisia and Algeria) (*SI Appendix*, Fig. S2). Similarly, three of the top ten countries with the highest consumption rates in 2023 were UMICs (Ecuador, Türkiye, and Serbia) compared to only two in 2016 (Ecuador and Türkiye) (*SI Appendix*, Fig. S2).

**Fig. 3.**
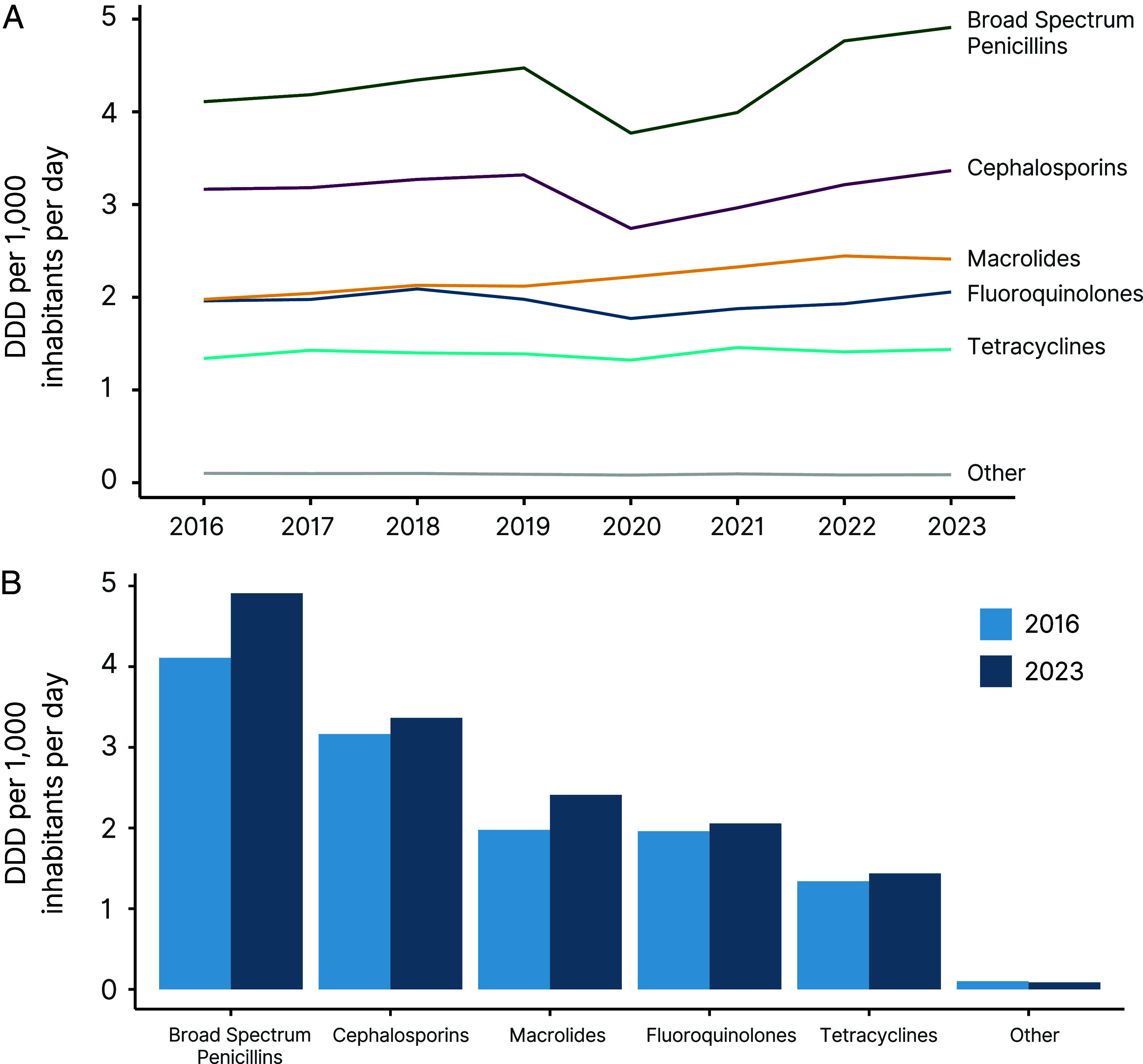
Global antibiotic consumption by antibiotic class. (*A*) Yearly changes in DDDs per 1,000 inhabitants per day for the top five antibiotic classes by consumption amount. All other classes were combined into other. (*B*) Antibiotic consumption differences between 2016 and 2023 for the top five antibiotic classes by consumption amount, with all other classes combined into other. DDD = defined daily dose. Data Source: Based on IQVIA MIDAS^®^ sales data for period 2016–2023. Copyright IQVIA. All rights reserved.

During the COVID-19 pandemic, the countries with the greatest declines in antibiotic consumption were the Philippines (−41.8%), Malaysia (−28.4%), Uruguay (−27.5%), Ecuador (−27.2%), and Argentina (−26.8%). As a group, MICs had the largest rebounds in 2021, led by Indonesia (22.8%), Argentina (18.6%), and South Africa (15.4%) for UMICs, and India (16.5%) and West Africa (15.3%) for LMICs. UMICs and HICs experienced the largest percentage increases in antibiotic consumption rates from 2021 to 2023 (17.1% and 21.7%, respectively). The COVID-19 pandemic had a limited impact on which types of drugs were consumed (Fig. 3). Broad-spectrum penicillins (BSPs), cephalosporins, macrolides, fluoroquinolones, and tetracyclines remained the classes with the highest consumption rates by a large margin.

**Fig. 4.**
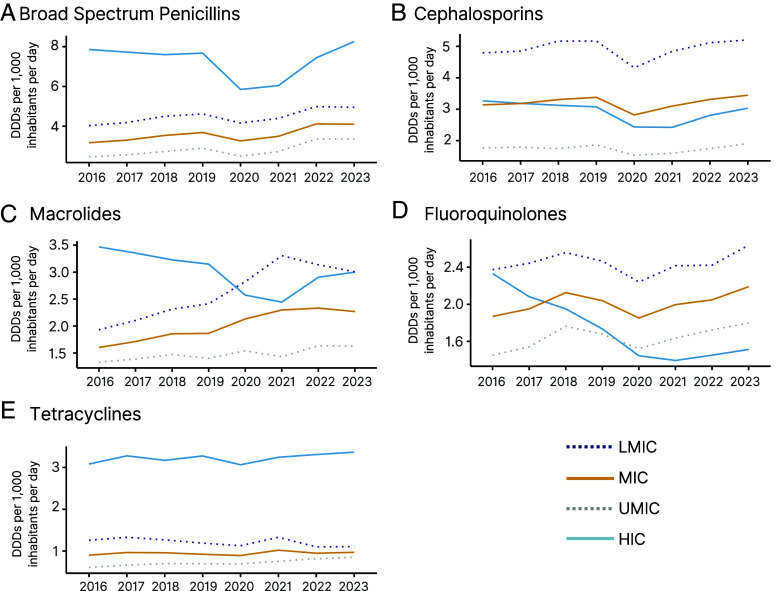
Global antibiotic consumption for the top five most consumed antibiotic classes by country income classification. Data are displayed as DDDs per 1,000 inhabitants per day. (*A*) BSP. (*B*) cephalosporins. (*C*) macrolides. (*D*) fluoroquinolones. (*E*) tetracyclines. Country income classifications noted as LMIC = lower-middle-income countries, MIC = middle-income countries, UMIC = upper-middle-income countries, HIC = high-income countries. Data Source: Based on IQVIA MIDAS^®^ sales data for period 2016–2023. Copyright IQVIA. All rights reserved.

Differences in the consumption rates by class and income group were apparent as well. The use of BSPs increased in all income classes throughout the study period (Fig. 4*A*), though during the pandemic, the largest decline was seen in HICs (−23.8%). Cephalosporin consumption rates in HICs were greater than that in MICs in 2016, but by the end of the study period, MICs had higher consumption, though the rates were over twice as high in LMICs as UMICs throughout the study period (mean, 1.7 vs. 4.9 DDDs per 1,000 inhabitants per day) (Fig. 4*B*). Macrolide use decreased sharply in 2020 in HICs but rose in MICs, driven largely by LMICs, and in 2020, macrolide use in LMICs surpassed that in HICs (2.8 compared to 2.6 DDDs per 1,000 inhabitants per day) (Fig. 4*C*). Fluoroquinolone consumption rates similarly started the period higher in HICs but by the end of the period had switched. However, in this case, the switch was largely due to use in HICs decreasing significantly. While consumption of “last-resort” antibiotics (carbapenems, oxazolidinones, glycylcyclines, and monobactams) remained at low levels relative to other antibiotic classes, MICs saw substantial percentage increases between 2016 and 2023: Carbapenem use increased 74.0%, oxazolidinone use increased 285.8%, and glycylcyclines use increased 221.5%. Only monobactam use decreased 82.0% (*SI Appendix*, Fig. S3).

**Fig. 5.**
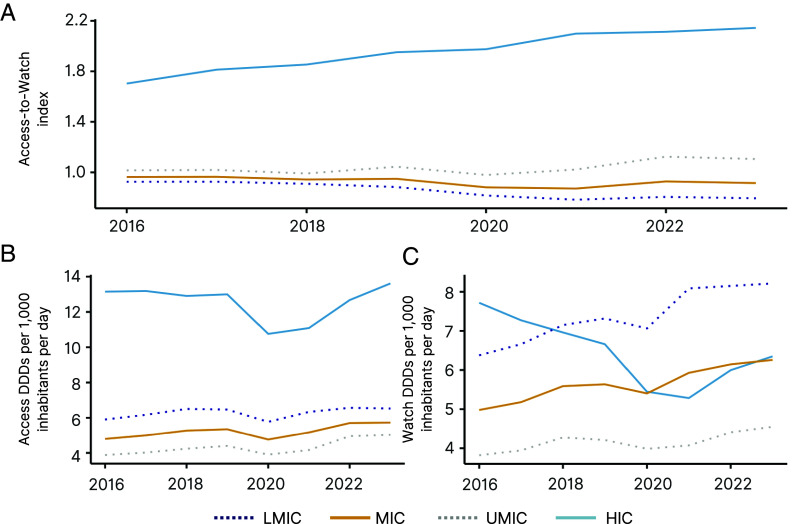
Global access and watch antibiotic consumption by country income classification. We illustrate (*A*) the Access-to-Watch index, as well as (*B*) Access and (*C*) Watch consumption by income classification for the period 2016–2023. The Access-to-Watch index is the ratio of Access to Watch consumption each year. Consumption data are displayed as DDDs per 1,000 inhabitants per day. Country income classifications noted as LMIC = lower-middle-income countries, MIC = middle-income countries, UMIC = upper-middle-income countries, HIC = high-income countries. Data Source: Based on IQVIA MIDAS^®^ sales data for period 2016–2023. Copyright IQVIA. All rights reserved.

Rates of consumption of Access and Watch antibiotics differed by income group, as well. HICs consumed consistently more Access antibiotics relative to Watch, with the Access-to-Watch index growing from 1.70 in 2016 to 2.14 in 2023 (Fig. 5*A*). MICs, on the other hand, consumed more Watch antibiotics than Access, with the Access-to-Watch index falling from 0.96 in 2016 to 0.92 in 2023. UMICs had a higher Access-to-Watch index than LMICs in all years. HICs outpaced MICs in Access consumption rates, as well, despite HIC Access consumption falling by 17.2% in 2020 (Fig. 5*B*). UMICs had the lowest Access consumption of all income groups. Watch consumption varied substantially by income group. While HICs had higher rates of Watch consumption than MICs in 2016 (7.7 vs. 5.0 DDDs per 1,000 inhabitants per day), in 2021, the MIC rate of 5.9 DDDs per 1,000 inhabitants per day surpassed the HIC rate of 5.3 DDDs per 1,000 inhabitants per day (Fig. 5*C*). The decrease in HIC Watch consumption preceded the onset of the COVID-19 pandemic and fell 29.5% from 2016 to 2020. The increase in MIC Watch consumption was driven largely by LMICs, whose rate of Watch consumption outpaced HICs by 2018 (7.2 vs. 7.0 DDDs per 1,000 inhabitants per day). In 2023, LMICs led all groups in Watch consumption at 8.2 DDDs per 1,000 inhabitants per day.

**Fig. 6.**
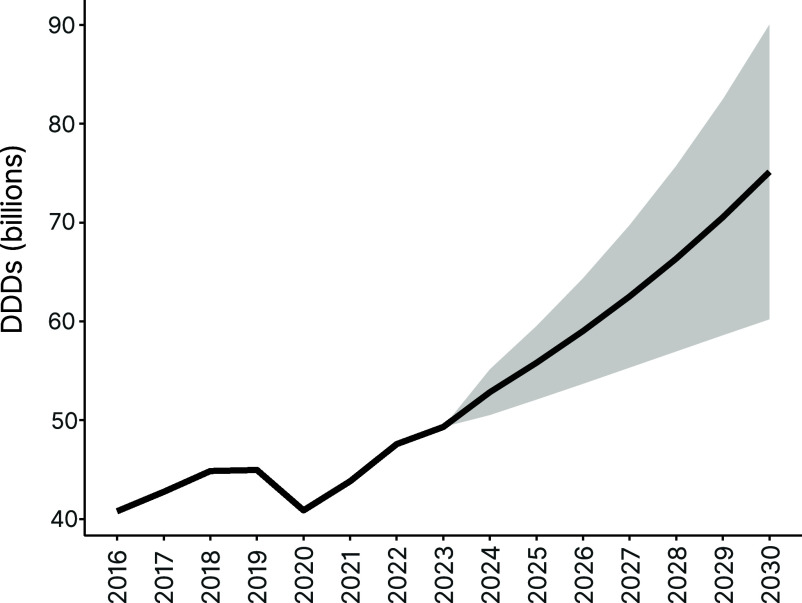
Estimated total global antibiotic consumption in DDD (billions). Global consumption estimates include totals for all countries in the database and use country income classification to estimate per capita use for countries not included. Per capita estimates were multiplied by each country’s estimated or forecasted population to generate estimated total. Line from 2023–2030 is the average projected change in antibiotic consumption assuming countries future rate of change is between their compounded annual growth rate from 2016 to 2019 and the rate from 2020–2023. The shaded region is the uncertainty range of the projection (includes only variance from projected rate of change). Data Source: Based on IQVIA MIDAS^®^ sales data for period 2016–2023. Copyright IQVIA. All rights reserved.

In 2023, estimated global antibiotic consumption (including estimates for countries for which data were unavailable) was 49.3 billion DDDs, or 17.0 DDDs per 1,000 inhabitants per day. This was an increase of 20.9% in total use and of 13.1% in the consumption rate from 2016, when global antibiotic consumption was estimated to be 40.8 billion DDDs, or 15.1 DDDs per 1,000 inhabitants per day. Assuming that future consumption in countries falls within current ranges of the study period, and assuming no changes in policy, our projections suggest that global antibiotic consumption could increase by 52.3% (uncertainty range [UR]: 22.1 to 82.6%) to a total of 75.1 (UR: 60.2 to 90.1) billion DDDs by 2030, and the antibiotic consumption rate could increase by 43.8% (UR: 15.2 to 72.3%) to 24.5 (19.6 to 29.4) DDDs per 1,000 inhabitants per day (Fig. 6).

## Discussion

Global antibiotic consumption in DDDs rose by 20.9% from 2016 to 2023, which was lower than the increase of 35.5% in the prior 7-y period (2008–2015) (4); however, a significant decrease occurred across all income groups in 2020 due to the COVID-19 pandemic, without which the total increase likely would have been higher. The relative impact of the pandemic on antibiotic use differed across countries, with the largest decreases taking place in HICs. In MICs, the largest declines were seen in those countries that had been increasing most rapidly prepandemic, reflecting the strong relationship between GDP growth and antibiotic use (4). During the pandemic, the vast majority of these countries maintained higher use than HICs and rebounded strongly in 2021, surpassing prior consumption rates. HICs experienced a “delayed rebound” in antibiotic consumption following the pandemic, with a very small (0.8%) increase in consumption in 2021 followed by larger increases in 2022 and 2023. While antibiotic consumption rates rose in 2023 in HICs, they had not quite returned to prepandemic levels by the end of 2023.

The delayed rebounds observed in HICs were likely due to longer adherence to policies and strategies to reduce transmission of SARS-CoV-2, such as mask wearing. As most antibiotic use in HIC occurs in the outpatient setting (18) and a significant fraction is driven by inappropriate use for respiratory infections (19), reductions in transmission of respiratory pathogens overall likely reduced antibiotic use. Another contributing factor to reduced transmission was likely an increase in individuals working from home. In higher-income economies, a larger fraction of jobs can be done at home (20), and though the pandemic officially ended in May 2023, working from home has remained at higher levels in HIC than prepandemic. What impact this may have on antibiotic use long term is worth further analysis.

Assessing antibiotic use at the national level is important because antimicrobial stewardship programs (ASPs) are often designed and implemented at that level. Several factors drive the rapid increase in antibiotic consumption in developing settings, including environmental, political, socioeconomic, and cultural factors (21, 22), though economic growth is likely the most important factor in lower-income settings. Recommendations to restrict antibiotic consumption must be context-specific as many policies intended to combat antimicrobial resistance (AMR) and avert antibiotic misuse/overuse have implications for sustainable development goals (SDGs). As countries grow economically and attempt to break out of the poverty cycle (SDG 1), they may be at risk for inappropriate antibiotic use, which could drive AMR and threaten progress toward healthcare goals (SDG 3) (23). Thus, increasing support for the development of national action plans and ASPs is necessary. However, equally important are the investments needed in measures to prevent infection (23, 24). Improvements in sanitation and hygiene measures, including the widespread implementation of water treatment facilities, sewer systems, and personal hygiene practices, played a pivotal role in significantly reducing the burden of infectious diseases and increasing life expectancies in HICs in the early 1900s. Many growing economies are facing rapid urbanization and population growth associated with higher population density and promotion of infectious disease spread (25, 26), which a focus on access to sanitation could alleviate.

In addition, increasing use of vaccines and point-of-care diagnostics could substantially decrease unnecessary antibiotic use, particularly in resource-limited environments. Such a reduction can occur directly, through vaccination against bacterial infections, such as pneumococcal conjugate vaccine, and indirectly, by diminishing the prevalence of viral illnesses that are frequently and inappropriately treated with antibiotics. Several recent studies have highlighted how vaccines (against bacterial pathogens and viruses) can help curb AMR and avert antibiotic prescriptions (27–31) and highlight the potential and importance of robust immunization programs and proper diagnostics as an AMR mitigation strategy, particularly in resource-poor settings.

However, investments and a focus on prevention do not diminish the need to reduce the misuse and overuse of antibiotics globally. A particularly useful mechanism is the World Health Organization’s Access, Watch and Reserve (AWaRe) framework (32, 33). AWaRe categories delineate which drugs should be prioritized for widespread access and which should be more strictly deployed. Crafting national action plans and ASPs with AWaRe at the forefront has the potential to preserve the power of antibiotics as a common, global resource and aligns with the theme of sustainable development. Cooperation regarding antibiotic stewardship and access, which has been major a focus of the 2024 United Nations General Assembly (UNGA) High-level Meeting on AMR, is crucial for the success of a global framework (34, 35). As shown in this analysis, Watch group antibiotic consumption relative to Access group antibiotic consumption in MICs, particularly LMICs, has increased over the period 2016–2023. Further study on the extent of the impact of the framework on global antibiotic consumption since its release in 2017 is warranted. This is especially important considering that the 2024 UNGA AMR High-level meeting declaration has set a target of increasing Access group antibiotic consumption to at least 70% of human antibiotic consumption globally by 2030 (35).

In addition, strengthening regulatory agencies and ministries of health in MICs is critical to improving antibiotic stewardship in these countries. The diverging trend in fluoroquinolone use between HICs and MICs highlights this issue as HICs steady decrease in use was due to regulatory agency warnings against fluoroquinolone use for risk of disabling and potentially permanent side effects (36, 37). The fact that use did not decline, but continued to increase in MICs, could lead to increased rates of adverse events potentially impacting public health and healthcare systems and exacerbating existing health disparities within these countries. Additionally, given the widespread unregulated use of antibiotics in many countries [e.g., without prescription (38)], educational campaigns in MICs advising against the use of this class of drugs for empiric therapy and respiratory infections need to target pharmacies and consumers as well as providers. The need for strengthening the capacity of regulatory agencies in MICs is also indicated by the rapid increases seen in the use of last-resort antibiotics (carbapenems, oxazolidinones, glycylcyclines, and monobactams). While rising resistance rates in many MIC countries suggest a need for access to more effective antibiotics, this must be balanced with increased regulation to prevent overuse and misuse.

Our analysis has some limitations. First, IQVIA MIDAS data were available for only 67 countries, most of which are HICs and UMICs. Thus, results may not be representative of all nations categorized by income levels, and not all countries had data categorized by sector, which may have impacted the total DDDs for drugs that have both an oral and intravenous formulations. Furthermore, data provided were estimated sales in kilograms, which may not accurately reflect consumption. Second, while identifying the burden of diseases requiring antibiotics provides a good baseline for measuring antibiotic overuse, the data utilized were aggregated sales data, which limits the ability to draw conclusions on overuse or misuse due to the absence of reasons for prescribing. Third, data only included human consumption; a more comprehensive One Health approach that includes surveillance of animal antibiotic consumption and agricultural use is needed to more effectively combat overuse and detect concerning AMR trends (39). Fourth, changes in countries’ economic categorization make it difficult to fully compare LMICs and UMICs to prior analyses—that is why we combined most analyses as MICs. An analysis of the data using income classifications from 2007 [2007 classifications were used in the previous analysis of data from 2000 to 2015 (4)] found that the major differences in the current paper were due largely to switches between class and not differences in consumption (*SI Appendix*, Fig. S4).

In conclusion, our findings show that while the COVID-19 pandemic had a major impact on antibiotic use across all income levels, the overarching trend of increasing global antibiotic consumption fueled by economic development in MICs remains. Our estimate of 49.3 billion DDDs for global use in 2023 was lower than our prior forecast for 2023 of 70.3 billion DDDs which assumed increasing annual growth rates. However, while the results were only slightly higher than our forecast assuming convergence toward a global median, because growth was interrupted by the pandemic, it is difficult to evaluate whether efforts to curb antibiotic use over the last decade have been effective. Furthermore, the rapid increases postpandemic suggest that the trajectory of growth may be continuing to increase. Thus, policies are urgently needed to promote increased antibiotic access and ensure that antibiotics are not misused or overused in the process. In addition, greater emphasis is needed globally on preventing transmission of infections to reduce the need for antibiotics, particularly the most effective drugs. Improving infrastructure and access to water, sanitation, and hygiene, particularly in rapidly developing nations, along with improved access to vaccination should be an important pillar in the fight against AMR.

## Materials and Methods

Data were retrieved for 67 countries from the IQVIA MIDAS® database, which provides estimated sales data for pharmaceutical drugs. IQVIA MIDAS® data are collected on a monthly basis from a sample of pharmacies and other outlets through which antibiotics and pharmaceutical drugs are dispensed. Each country’s data are split by year and sector (hospital and retail). In total, we used data from 67 countries over the period 2016–2023. See Table 1 for included countries and their data availability.

**Table 1.** Country data available by sector

Country	Retail	Hospital	Country	Retail	Hospital
Algeria	Yes	No	Malaysia	Yes	No
Argentina	Yes	No	Mexico	Yes	No
Australia	Yes	Yes	Morocco	Yes	No
Austria	Yes	Yes	Netherlands	Yes	Yes
Belarus	Yes	Yes	New Zealand	Yes	Yes
Belgium	Yes	Yes	Norway	Yes	Yes
Brazil	Yes	Yes	Pakistan	Yes	No
Bulgaria	Yes	Yes	Peru	Yes	No
Canada	Yes	Yes	Philippines	Yes	Yes
Central America^*^	Yes	No	Poland	Yes	Yes
Chile	Yes	No	Portugal	Yes	Yes
China	No	Yes	Puerto Rico	Yes	Yes
Colombia	Yes	No	Romania	Yes	Yes
Croatia	Yes	Yes	Russia	Yes	Yes
Czech Republic	Yes	Yes	Saudi Arabia	Yes	Yes
Denmark	Yes	Yes	Serbia	Yes	No
Ecuador	Yes	No	Slovakia	Yes	Yes
Egypt^†^	Yes	Yes	Slovenia	Yes	No
Finland	Yes	Yes	South Africa	Yes	No
France	Yes	Yes	South Korea	Yes	Yes
Germany	Yes	Yes	Spain	Yes	Yes
Greece	Yes	No	Sweden	Yes	Yes
Hong Kong	Yes	No	Switzerland	Yes	Yes
Hungary	Yes	Yes	Taiwan	Yes	Yes
India	Yes	No	Thailand	Yes	Yes
Indonesia	Yes	No	Tunisia	Yes	Yes
Ireland	Yes	No	Türkiye	Yes	Yes
Italy	Yes	Yes	United Arab Emirates	Yes	No
Japan	Yes	Yes	United Kingdom	Yes	Yes
Jordan	Yes	No	United States	Yes	Yes
Kuwait	Yes	No	Uruguay	Yes	No
Latvia	Yes	No	Vietnam	Yes	Yes
Lebanon	Yes	No	West Africa^‡^	Yes	No
Luxembourg	Yes	No			

Data Source: Based on IQVIA MIDAS^®^ sales data for period 2016–2023. Copyright IQVIA. All rights reserved.

^*^Central America includes Guatemala, Honduras, El Salvador, Nicaragua, Costa Rica, and Panama.

^†^Egypt did not have hospital-level data for Q1 2016–Q2 2018, so data were interpolated based on the ratio for 2023.

^‡^West Africa includes Benin, Burkina Faso, Cameroon, Chad, Republic of the Congo, Gabon, Guinea, Ivory Coast, Mali, Niger, Senegal, and Togo.

Antibiotic sales data were obtained in estimated kilograms per active ingredient and converted to DDDs utilizing the Anatomical Therapeutic Chemical Classification System (ATC/DDD) published by the WHO Collaborating Centre for Drug Statistics Methodology following protocols from prior study (4, 40). We accounted for the different sectors in our data by assuming that hospital antibiotics were administered parenterally (intravenously) and retail antibiotics were administered orally similar to ref. 4. DDD unit values by route of administration were obtained from the ATC/DDD index for all molecules (39). Combination drugs were broken into their individual molecules, and one main molecule was determined. For molecules in the IQVIA MIDAS data without defined DDDs, we estimated them from other sources. See *SI Appendix*, Tables S2–S4 and *Supplementary Text* for all included DDD unit values and sources which were not in the ATC/DDD database. See Dataset S1 for consumption data in DDDs for each country.

Antibiotic consumption rates were measured in DDDs per 1,000 inhabitants per day, utilizing population estimates retrieved from World Bank and country government data (41, 42). Consumption rates among groups of countries based on their World Bank income classification in 2023 were subsequently compared. The World Bank income classifications used in this study are LMIC (n = 11), UMIC (n = 17), and HIC (n = 39) for 2023 (43). West Africa was designated LMIC as that was the most reflective of the aggregate; Central America was designated UMIC as that was most reflective of the aggregate. See *SI Appendix*, Table S5 for the list of countries and their income classifications used in the analysis, as well as their 2007 income classifications which were used in the previous analysis (4).

Antibiotics and combinations were classified as either “Access” or “Watch” drugs per the most recent (2023) WHO AWaRe framework for further analysis (44). The Access-to-Watch index for a given year was calculated by dividing Access consumption by Watch consumption for that year. To evaluate the impact of the COVID-19 pandemic on antibiotic consumption, we conducted an ITSA. ITSAs are a type of study design often used in public health to evaluate the impact of an intervention (here, the onset of the COVID-19 pandemic) on an outcome of interest (here, antibiotic consumption rates) (45, 46). We conducted a separate ITSA for each income group on its annual antibiotic consumption rate from 2016 to 2023. The “intervention” point was set to the beginning of 2020 such that 2020 data were included in the “postintervention” period. Regressions were calculated using a generalized least squares model by maximum likelihood; we confirmed series stationarity and accounted for autocorrelation for each income group through autoregressive and moving average adjustments. See *SI Appendix*, Supplemental Text** for extended methodology for the ITSAs.

Global antibiotic consumption was calculated by extrapolating antibiotic use for countries not included in the IQVIA data. Extrapolations were based on the average per capita antibiotic use for countries with data in the same income group. Low-income countries were grouped with LMICs in our analysis. To project global antibiotic use through 2030, we conducted a sensitivity analysis assuming that countries’ growth rates ranged between each countries’ compounded annual growth rate from 2016 to 2019 and their compounded annual growth rate from 2020 to 2023. For extrapolated countries, we used the income group’s growth rates. We used a triangular distribution with the peak midway between the two growth rates on the assumption that prior growth was a predictor for future growth. The mean and variance were calculated by calculating the projected change in antibiotic use in total DDDs for the entire distribution stratified into 1,000 segments. The uncertainty range was calculated as the SD of the variance. Stata 16.1, R version 4.3.2, and Microsoft Excel were used for cleaning, analyses, and data visualizations.

The statements, findings, conclusions, views, and opinions contained and expressed in this article are based in part on data obtained under license from the following: IQVIA MIDAS® sales data for the period 2016–2023: quarterly-country level sales of antibiotic sales (ATC3: J01). Geography: Global (Algeria, Argentina, Australia, Austria, Belarus, Belgium, Bulgaria, Canada, Chile, China, Colombia, Costa Rica, Croatia, Czech, Denmark, Ecuador, Egypt, El Salvador, Finland, France, Germany, Greece, Guatemala, Honduras, Hong Kong, Hungary, India, Indonesia, Ireland, Italy, Japan, Jordan, Kuwait, Latvia, Lebanon, Luxembourg, Malaysia, Mexico, Morocco, Netherlands, New Zealand, Nicaragua, Norway, Pakistan, Panama, Peru, Philippines, Poland, Portugal, Puerto Rico, Romania, Russia, Saudi Arabia, Serbia, Slovakia, Slovenia, South Africa, South Korea, Spain, Sweden, Switzerland, Taiwan, Thailand, Tunisia, Türkiye, United Arab Emirates, United Kingdom, United States, Uruguay, Vietnam, West Africa); Measures: DDDs, calculated by GSK using IQVIA MIDAS Volume Data, reflecting estimates of real world activity. Copyright IQVIA. All rights reserved. The statements, findings, conclusions, views, and opinions contained and expressed herein are not necessarily those of IQVIA.

## Supplementary Material

Appendix 01 (PDF)

Dataset S01 (XLSX)

## Data Availability

All study data are included in the article and/or supporting information.
