# Supplementary material for: Global trends in antibiotic consumption during 2016–2023 and future projections through 2030
Source: Proc Natl Acad Sci U S A. 2024 Nov 18;121(49):e2411919121. doi: 10.1073/pnas.2411919121 (PMC11626136; doi:10.1073/pnas.2411919121)
Supplement: Supplementary file 1 — Appendix 01 (PDF) [file pnas.2411919121.sapp.pdf]

Supporting Information for

Global trends in antibiotic consumption during 2016–2023 and future projections through 2030

Eili Y. Klein, Isabella Impalli, Suprena Poleon, Philippe Denoel, Mariateresa Cipriano, Thomas P. Van Boeckel, Simone Pecetta, David E. Bloom, Arindam Nandi

This PDF file includes:

Figures S1 to S4
Tables S1 to S5
Supplementary text
Legend for Dataset S1
SI References

Other supporting materials for this manuscript include the following:
Dataset S1

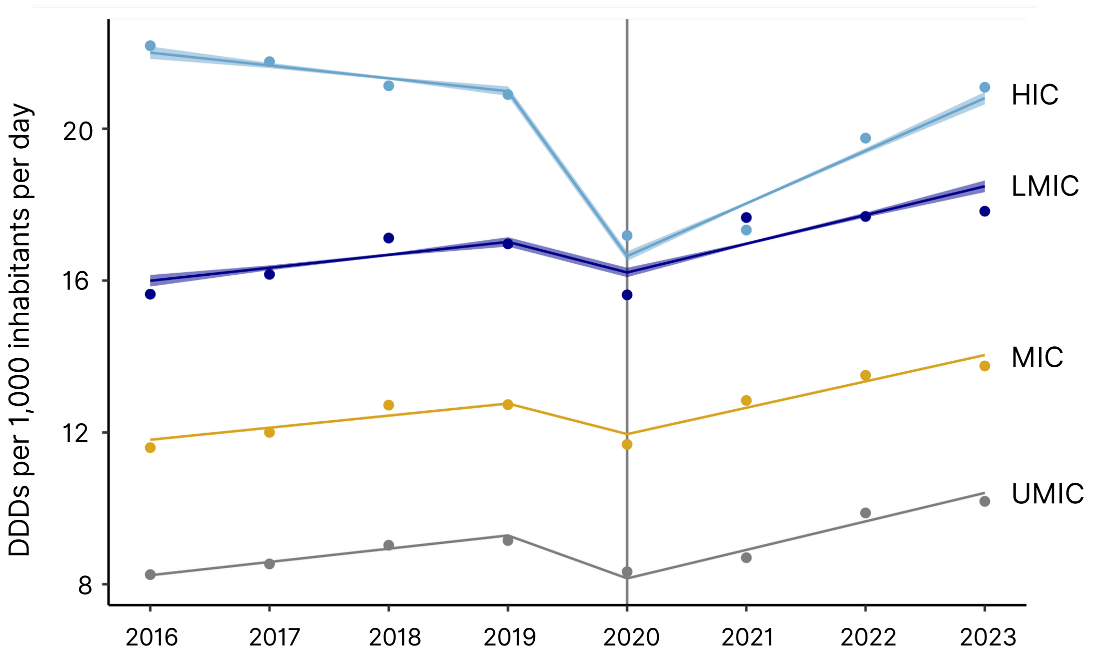

Income	β_0 Base Intercept	β_1 Time	β_2 Intervention	β_3 Time Post-Intervention
**HIC**	22.00 (0.08)	-0.34 (0.05)	-5.73 (0.17)	1.73 (0.03)
	p = 0.0000	p = 0.0022	p = 0.0000	p = 0.0000
**LMIC**	15.99 (0.08)	0.34 (0.05)	-1.56 (0.17)	0.42 (0.03)
	p = 0.0000	p = 0.0018	p = 0.0007	p = 0.0002
**MIC**	11.81 (0.01)	0.32 (0.00)	-1.50 (0.01)	0.38 (0.00)
	p = 0.0000	p = 0.0000	p = 0.0000	p = 0.0000
**UMIC**	8.24 (0.00)	0.35 (0.00)	-1.88 (0.00)	0.40 (0.00)
	p = 0.0000	p = 0.0000	p = 0.0000	p = 0.0000

Figure S1 and Table S1. Interrupted time series analysis results. We illustrate the measured rate (data points), generalized least squares regression (line), and 95% confidence intervals (shaded regions) for annual antibiotic consumption in each income group in defined daily doses (DDDs) per 1,000 inhabitants per day. The table contains model coefficients, along with their standard error (in parentheses) and significance values (p). For MIC and UMIC, confidence intervals were too small to be seen on the graph (see **Table S1** for standard error values). Error was very low due to the small number of data points and ARMA adjustments. See Supplementary text for extended methodology. Country income classifications noted as: LMIC = lower-middle-income countries, UMIC = upper-middle-income countries, MIC = middle-income countries, HIC = high-income countries.

Data Source: Based on IQVIA MIDAS® sales data for period 2016–2023. Copyright IQVIA. All rights reserved.

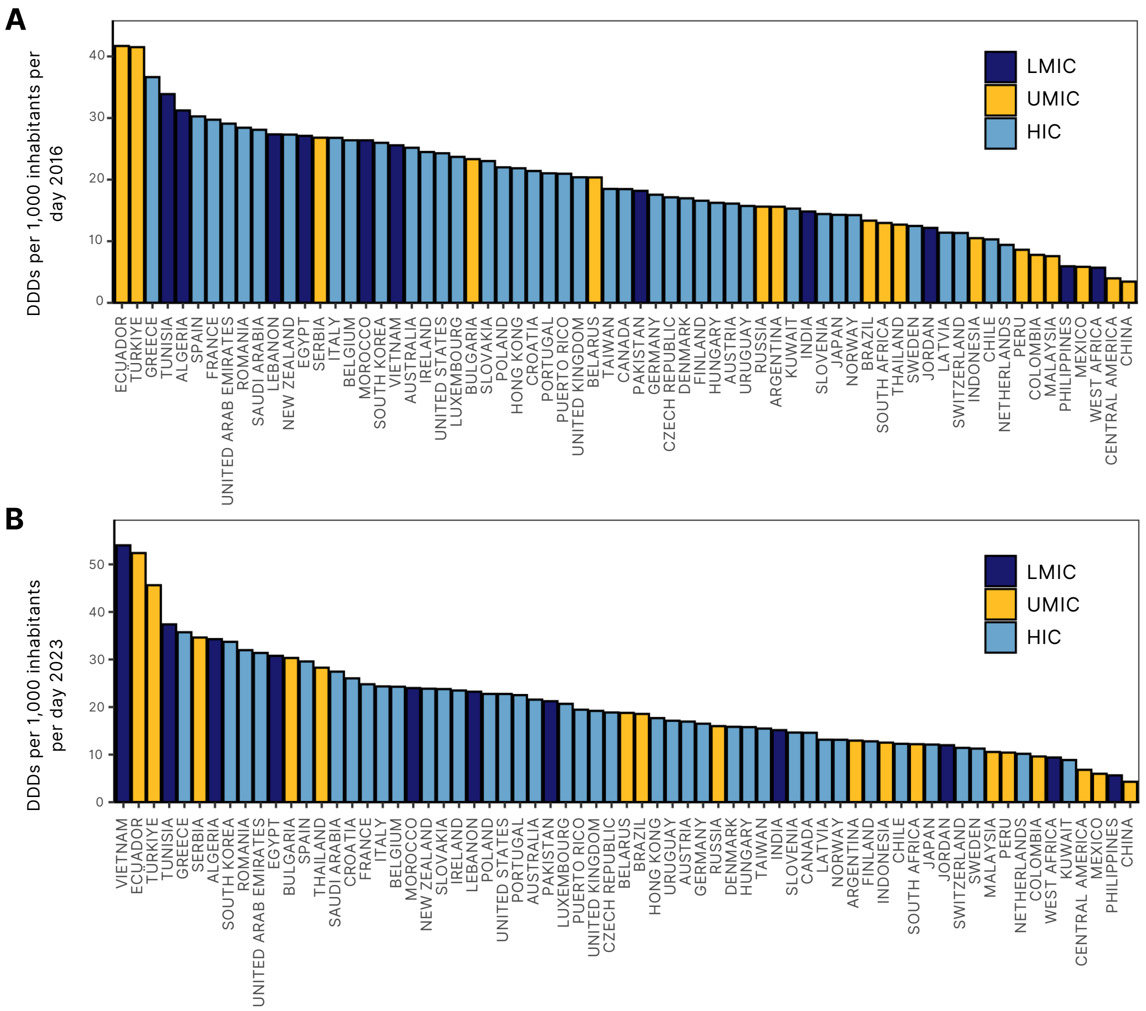

Figure S2. A) Antibiotic consumption rate by country for 2016 in defined daily doses per 1,000 inhabitants per day. B) Antibiotic consumption rate by country for 2023 in defined daily doses per 1,000 inhabitants per day. LMIC = lower-middle-income countries, UMIC = upper-middle-income countries, HIC = high-income countries, DDD = defined daily dose.

Data Source: Based on IQVIA MIDAS® sales data for period 2016–2023. Copyright IQVIA. All rights reserved.

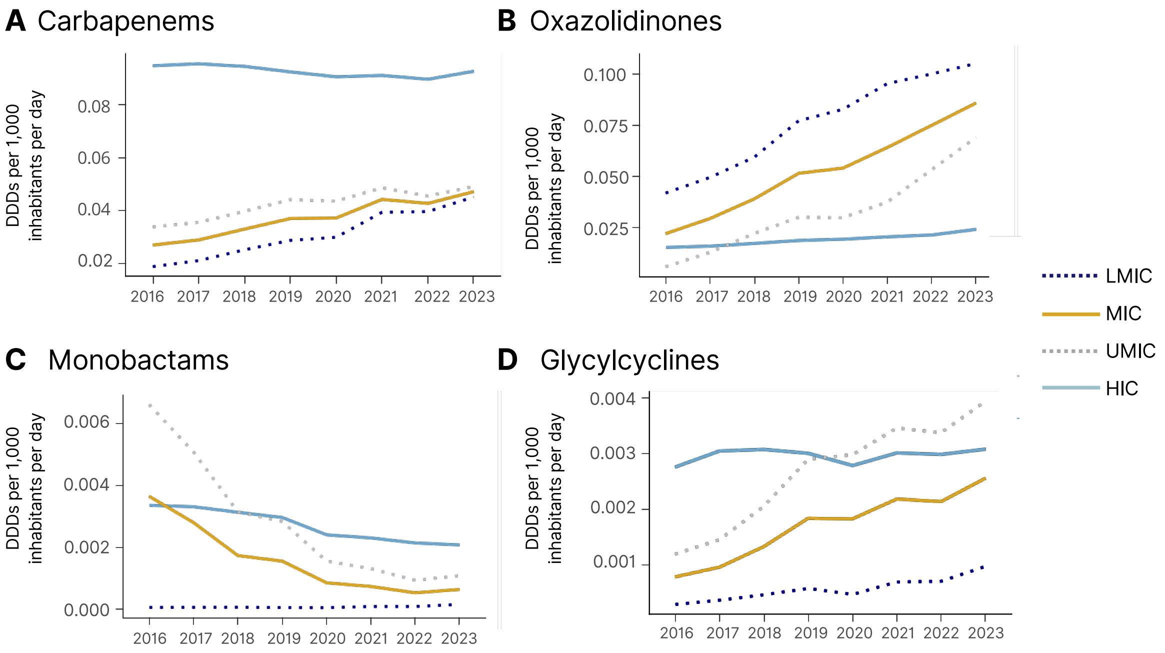

Figure S3. Global antibiotic consumption for the top four last resort antibiotic classes by country income classification. Data are displayed as defined daily doses (DDDs) per 1,000 inhabitants per day. (A) carbapenems. (B) oxazolidinones. (C) monobactams. (D) glycylcyclines. Country income classifications noted as: LMIC = lower-middle-income countries, MIC = middle-income countries, UMIC = upper-middle-income countries, HIC = high-income countries.

Data Source: Based on IQVIA MIDAS® sales data for period 2016–2023. Copyright IQVIA. All rights reserved.

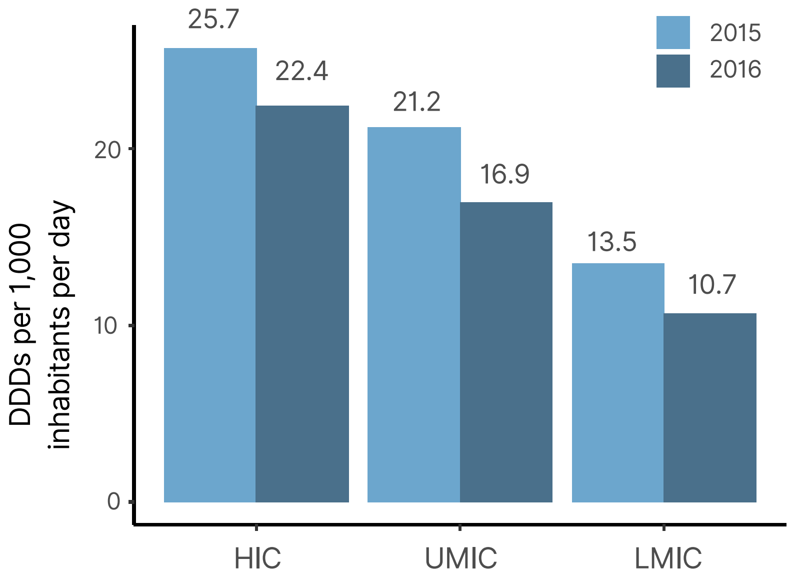

Figure S4. 2015 and 2016 DDDs per 1,000 inhabitants per day, calculated using 2007 income groupings from the World Bank, in alignment with previous analysis (1). 2015 data are from the previous analysis (1). See Table S6 for complete list of groupings used in current analysis and the changes since previous analysis. LMIC = lower-middle-income countries, UMIC = upper-middle-income countries, HIC = high-income countries, DDD = defined daily dose.

Data Source: Based on IQVIA MIDAS® sales data for period 2016–2023. Copyright IQVIA. All rights reserved.

Table S2. DDDs for molecules without value in the ATC/DDD 2023 database where other source was used.

Antibiotic	Route	DDD (g)	Source
Acetyl Kitasamycin	Oral	1.2	(2)
Antofloxacin	Oral	0.5	(1)
Antofloxacin	Parenteral	0.3	(3)
Bacitracin	Oral	0.68	(1)
Balofloxacin	Oral	0.2	(2)
Cefathiamidine	Parenteral	4	(4)
Cefiderocol*	Parenteral	6	(5)
Cefiderocol	Oral	6	(5)
Cefoselis	Parenteral	3.3	(1)
Contezolid	Parenteral	1.6	(6)
Contezolid	Oral	1.6	(6)
Eravacycline*	Parenteral	0.14	(7)
Eravacycline	Oral	0.14	(7)
Etimicin	Parenteral	0.5	(1)
Faropenem	Parenteral	0.9	(1)
Furbenicillin	Oral	1.5	(1)
Kitasamycin	Oral	1.2	(2)
Lefamulin	Parenteral	0.3	(8)
Lefamulin	Oral	1.2	(8)
Levonadifloxacin	Oral	2	(9)
Levonadifloxacin	Parenteral	1.6	(9)
Meleumycin	Parenteral	1.3	(1)
Meleumycin	Oral	1.3	(1)
Micronomicin	Parenteral	0.24	(2)
Norvancomycin	Parenteral	1.3	(1)
Norvancomycin	Oral	2	(1)
Oritavancin	Parenteral	1.2	(10)
Telavancin	Parenteral	1.3	(1)
Telavancin	Oral	2	(1)

Abbreviations: ATC/DDD = Anatomical Therapeutic Chemical Classification System, DDD = defined daily dose
* Note that cefiderocol and eravacycline parenteral were added to the ATC/DDD database in 2024 with the same values listed here.

Table S3. DDDs for molecules without value in the ATC/DDD 2023 database where DDD was calculated as an average of antibiotic class, by route or location, from the ATC/DDD database (11).

Antibiotic	Route	DDD (g)
Etimicin	Oral	2.8
Guamecycline	Parenteral	0.54
Guamecycline	Oral	0.64
Metronidazole	Oral	0.85
Sulbactam	Oral	0.85

Abbreviations: ATC/DDD = Anatomical Therapeutic Chemical Classification System, DDD = defined daily dose

Table S4. DDDs for molecules and routes of administration without value in the ATC/DDD 2023 database where DDD was calculated as an average of molecule values by other routes of administration from the ATC/DDD database (11) or values from Table S1.

Antibiotic	Route	DDD (g)	Antibiotic	Route	DDD (g)
Amikacin	Oral	1	Isepamicin	Oral	0.4
Arbekacin	Oral	0.2	Josamycin	Parenteral	2
Aztreonam*	Oral	4	Kanamycin	Oral	1
Bacitracin	Oral	0.68	Kitasamycin	Parenteral	1.2
Biapenem	Oral	1.2	Lomefloxacin	Parenteral	0.4
Carbenicillin	Oral	12	Mecillinam	Oral	1.2
Cefadroxil	Parenteral	2	Meropenem	Oral	3
Cefalexin	Parenteral	2	Metacycline	Parenteral	0.6
Cefazolin	Oral	3	Micronomicin	Oral	0.24
Cefdinir	Parenteral	0.6	Nafcillin	Oral	3
Cefditoren Pivoxil	Parenteral	0.4	Neomycin	Parenteral	5
Cefepime	Oral	2	Norfloxacin	Parenteral	0.8
Cefixime	Parenteral	0.4	Oleandomycin	Parenteral	1
Cefotaxime	Oral	4	Oritavancin	Oral	1.2
Cefotetan	Oral	4	Panipenem	Oral	2
Cefpirome	Oral	4	Paromomycin	Parenteral	3
Cefpodoxime Proxetil	Parenteral	0.4	Penicillin G	Oral	3.6
Ceftazidime	Oral	4	Penicillin V	Parenteral	2
Ceftizoxime	Oral	4	Pheneticillin	Parenteral	1
Ceftriaxone	Oral	2	Pivmecillinam	Parenteral	0.6
Chlortetracycline	Parenteral	1	Pristinamycin	Parenteral	2
Clofoctol	Parenteral	1.5	Ribostamycin	Oral	1
Clofoctol	Oral	1.5	Rifabutin	Parenteral	0.15
Clometocillin	Parenteral	1	Rifamycin	Oral	0.6
Dalbavancin	Oral	1.5	Roxithromycin	Parenteral	0.3
Daptomycin	Oral	0.28	Sisomicin	Oral	0.24
Demeclocycline	Parenteral	0.6	Spectinomycin	Oral	3
Dibekacin	Oral	0.14	Spiramycin	Parenteral	3
Dirithromycin	Parenteral	0.5	Streptomycin	Oral	1
Doripenem	Oral	1.5	Tebipenem	Parenteral	0.56
Enoxacin	Parenteral	0.8	Teicoplanin	Oral	0.4
Ertapenem	Oral	1	Telithromycin	Parenteral	0.8
Erythromycin Stinoprate	Parenteral	1	Temocillin	Oral	4
Gemifloxacin	Parenteral	0.32	Ticarcillin	Oral	15
Gentamicin	Oral	0.24	Tigecycline	Oral	0.1
Imipenem	Oral	2	Tobramycin	Oral	0.24

Abbreviations: ATC/DDD = Anatomical Therapeutic Chemical Classification System, DDD = defined daily dose
* For aztreonam calculation, we did not include the DDD for the inhalable solution

Table S5. World Bank income classifications as of 2023 for countries used in analysis compared to 2007 groupings.

**Country**	**2023** (12)	**2007**[a]
Algeria	LMIC	UMIC
Argentina	UMIC	UMIC
Australia	HIC	HIC
Austria	HIC	HIC
Belarus	UMIC	N/A[b]
Belgium	HIC	HIC
Brazil	UMIC	UMIC
Bulgaria	UMIC	UMIC
Canada	HIC	HIC
Central America	UMIC	N/A[c]
Chile	HIC	UMIC
China	UMIC	LMIC
Colombia	UMIC	LMIC
Croatia	HIC	UMIC
Czech Republic	HIC	HIC
Denmark	HIC	HIC
Ecuador	UMIC	LMIC
Egypt	LMIC	LMIC
Finland	HIC	HIC
France	HIC	HIC
Germany	HIC	HIC
Greece	HIC	HIC
Hong Kong	HIC	HIC
Hungary	HIC	HIC
India	LMIC	LMIC
Indonesia	UMIC	LMIC
Ireland	HIC	HIC
Italy	HIC	HIC
Japan	HIC	HIC
Jordan	LMIC	LMIC
Kuwait	HIC	HIC
Latvia	HIC	UMIC
Lebanon	LMIC	UMIC
Luxembourg	HIC	HIC
Malaysia	UMIC	UMIC
Mexico	UMIC	UMIC
Morocco	LMIC	LMIC
Netherlands	HIC	HIC
New Zealand	HIC	HIC
Norway	HIC	HIC
Pakistan	LMIC	LMIC
Peru	UMIC	LMIC
Philippines	LMIC	LMIC
Poland	HIC	HIC
Portugal	HIC	HIC
Puerto Rico	HIC	HIC
Romania	HIC	UMIC
Russia	UMIC	UMIC
Saudi Arabia	HIC	HIC
Serbia	UMIC	UMIC
Slovakia	HIC	HIC
Slovenia	HIC	HIC

South Africa	UMIC	UMIC
South Korea	HIC	HIC
Spain	HIC	HIC
Sweden	HIC	HIC
Switzerland	HIC	HIC
Taiwan	HIC	HIC
Thailand	UMIC	LMIC
Tunisia	LMIC	LMIC
Türkiye	UMIC	UMIC
United Arab Emirates	HIC	HIC
United Kingdom	HIC	HIC
United States	HIC	HIC
Uruguay	HIC	UMIC
Vietnam	LMIC	LMIC
West Africa	LMIC	LMIC

Abbreviations: HIC = high-income countries, UMIC = upper-middle-income countries, LMIC = lower-middle-income countries

Shading indicates a change between this analysis and the previous analysis (1).

[a] Previous analysis (1) used income groupings from 2007.

[b] Belarus was not included in the previous analysis (1).

[c] Central America was not classified in the previous analysis (1).

* Central America includes Guatemala, Honduras, El Salvador, Nicaragua, Costa Rica, and Panama.

** West Africa includes Benin, Burkina Faso, Cameroon, Chad, Republic of the Congo, Gabon, Guinea, Ivory Coast, Mali, Niger, Senegal, and Togo.

Supplementary text. Extended methods.

Interrupted Time Series Analysis. For the interrupted time series, the linear model is of the form given in Equation S1 (13):

$$\widehat{C_t} = \widehat{\beta_0} + \widehat{\beta_1}t + D_t\left(\widehat{\beta_2} + \widehat{\beta_3}(t - t_i)\right) + \varepsilon_t \qquad \text{(Eq S1)}$$

Where C_t is consumption as a function of time t, t_i is the onset of the COVID-19 pandemic (2020), D_t is an indicator variable which is set to 0 before t_i and 1 beginning at t_i and thereafter, β_0 is the baseline level of consumption at the first time step, β_1 is the slope of the function before the pandemic, β_2 is the change in the baseline consumption level due to the pandemic (such that $\beta_0 + \beta_2$ is the new baseline), β_3 is the change in the slope of the function due to the pandemic (such that $\beta_1 + \beta_3$ is the new slope), and ε_t is a general error term.

We opted to use the annual data instead of quarterly data, which had a visually obvious seasonal trend that was difficult to extract with so few data points. For each income group, time series stationarity was confirmed via the Kwiatkowski–Phillips–Schmidt–Shin test (14) at a significance level of $\alpha = 0.05$. Autocorrelation of data was then addressed using a generalized autoregressive moving average (ARMA) model, with parameters p (autoregressive) and q (moving average) chosen empirically by ranging pairwise values between 0 and 4 and choosing the combination with the lowest Akaike Information Criterion (AIC) and Bayesian Information Criterion (BIC) for each income classification. The interrupted time series regression was performed using generalized least squares (GLS) model fit by maximum likelihood with the autocorrelation adjustments as described. GLS was chosen over ordinary least squares to account for the autocorrelation observed (15).

DDD calculations for remaining values. The DDD in g for colistin was calculated as follows: colistin is 12,500 IU per mg of colistimethate sodium according to (16). In 1 g of colistimethate sodium, there is 12.5 million IU of colistin. According to the ATC/DDD index (11), the value for parenteral colistin is 9 million IU. We then divided 9 million by 12.5 million to derive the DDD value in g/kg for colistin (=0.72 g). The DDD in g for polymyxin B was calculated using the same methodology for a value of 0.15 g. Note that data for ofloxacin for Tunisia were excluded from analysis, and fluconazole (antifungal), clotrimazole (antifungal), and nitazoxanide (antiprotozoal) medications were omitted from analysis for all countries because they did not comply with the ATC3 classes used.

Dataset S1 (separate file). Dataset S1 reflects antibiotic consumption in DDDs disaggregated by country, antibiotic class, and year (2016–2023). Data were available for 67 'countries' as described in Table 1 in the main text.

Original Data Source: Based on IQVIA MIDAS® sales data for period 2016–2023. Copyright IQVIA. All rights reserved.

SI References

1. E. Y. Klein, *et al.*, Global increase and geographic convergence in antibiotic consumption between 2000 and 2015. *Proc Natl Acad Sci USA* **115**, E3463–E3470 (2018).
2. D.-S. Kim, N.-S. Kim, S.-H. Lee, Determination of defined daily dose of medicines using nominal group technique and analysis of antibiotics use in national insurance claim data: focused on antibiotics without DDD of WHO. *Korean Journal of Clinical Pharmacy* **17**, 19–32 (2007).
3. J. Wang, *et al*., Pharmacokinetics of antofloxacin hydrochloride in healthy male subjects after multiple intravenous dose administration. *Xenobiotica* **41**, 561-566 (2011).
4. W. Zhang, *et al.*, Antibiotic use in five children's hospitals during 2002–2006: the impact of antibiotic guidelines issued by the Chinese Ministry of Health. *Pharmacoepidemiology and Drug Safety* **17**, 306–311 (2008).
5. Shionogi, *Using Fetroja* (https://www.fetroja.com/using-fetroja#important-safety-information; accessed March 10, 2024).
6. S. Hoy, Contezolid, first approval. *Drugs* **81(**13)**,** 1587-1591 (2021).
7. S. Alosaimy, Evaluation of Eravacycline: A Novel Fluorocycline. *Pharmacotherapy: The Journal of Human Pharmacology and Drug Therapy* **40(**3)**,** 221-238 (2020).
8. European Medicines Agency., *Xenleta product information* (2021) (https://www.ema.europa.eu/en/medicines/human/EPAR/xenleta; accessed March 10, 2024).
9. Wockhardt., *Prescribing Information of Emrok & Emrok O* (2022) (https://emrok.co.in/Prescribing-Information.html; accessed March 10, 2024).
10. Melinta Therapeutics, Inc., *Orbactiv Prescribing Information* (2022) (https://www.orbactiv.com/pdfs/orbactiv-prescribing-information.pdf; accessed January 5, 2024).
11. World Health Organization, *ATC/DDD Index 2023* (2023) (https://www.whocc.no/atc_ddd_index/; accessed April 26, 2023).
12. World Bank, World Bank Country and Lending Groups. (https://datahelpdesk.worldbank.org/knowledgebase/articles/906519; accessed April 4, 2024).
13. S. L. Turner, et al., Comparison of six statistical methods for interrupted time series studies: empirical evaluation of 190 published series. BMC Medical Research Methodology 21, 134 (2021).
14. M. Poulos, Determining the Stationarity distance via a reversible stochastic process. *PloS one* **11**, e0164110 (2016).
15. J. L. Bernal, S. Cummins, A. Gasparrini, Interrupted time series regression for the evaluation of public health interventions: a tutorial. International journal of epidemiology 46, 348–355 (2017).
16. Datapharm Ltd, Electronic Medicines Compendium (https://www.medicines.org.uk/emc; accessed January 5, 2024).
